# Epigenetic Regulation of Endothelial Dysfunction and Inflammation in Pulmonary Arterial Hypertension

**DOI:** 10.3390/ijms222212098

**Published:** 2021-11-09

**Authors:** Jaylen Hudson, Laszlo Farkas

**Affiliations:** Department of Internal Medicine, Division of Pulmonary Disease, Critical Care and Sleep Medicine, Davis Heart & Lung Research Institute, The Ohio State University, Columbus, OH 43210, USA; hudson.607@buckeyemail.osu.edu

**Keywords:** pulmonary arterial hypertension, endothelial cell, endothelial dysfunction, inflammation, epigenetic regulation

## Abstract

Once perceived as a disorder treated by vasodilation, pulmonary artery hypertension (PAH) has emerged as a pulmonary vascular disease with severe endothelial cell dysfunction. In the absence of a cure, many studies seek to understand the detailed mechanisms of EC regulation to potentially create more therapeutic options for PAH. Endothelial dysfunction is characterized by complex phenotypic changes including unchecked proliferation, apoptosis-resistance, enhanced inflammatory signaling and metabolic reprogramming. Recent studies have highlighted the role of epigenetic modifications leading to pro-inflammatory response pathways, endothelial dysfunction, and the progression of PAH. This review summarizes the existing literature on epigenetic mechanisms such as DNA methylation, histone modifications, and non-coding RNAs, which can lead to aberrant endothelial function. Our goal is to develop a conceptual framework for immune dysregulation and epigenetic changes in endothelial cells in the context of PAH. These studies as well as others may lead to advances in therapeutics to treat this devastating disease.

## 1. Introduction

Pulmonary Arterial Hypertension (PAH) is defined by abnormally high blood pressure, increased pulmonary vascular resistance, and pulmonary arteriopathy. This life-threatening disease can result in progressive symptoms such as shortness of breath, right heart failure, and death. Although the exact cause remains unknown, many studies seek to improve the poor prognosis and limited therapeutic options of the disease. One of the major limitations of currently approved PAH treatments is that these therapies mainly target the vasotonus which is not sufficient in mitigating the occlusive arteriopathy in PAH. Endothelial cells (ECs) have become a focal point in PAH pathophysiology due to their critical roles in lung vascular function [1]. The disruption of EC function can occur by cardiovascular risk factors which are known to promote oxidative stress and other metabolic changes [2,3,4]. Endothelial dysfunction manifests with features, such as as a cancer cell-like phenotype including unchecked proliferation, apoptosis-resistance, increased pro-inflammatory signaling, and metabolic reprogramming towards glycolysis [5,6]. These unfavorable changes in ECs are often compounded with other irregularities such as altered hemostasis and thrombosis [7,8]. Injured ECs also undergo endothelial-to-mesenchymal transition (EnMT) which severely impacts their functional characteristics [9]. EnMT ECs show a pro-inflammatory phenotype and altered blood barrier function [9]. A more comprehensive understanding of the mechanisms leading to endothelial dysfunction will aid in designing specific approaches to treat PAH as well as other pulmonary diseases [10].

The observation that inflammation is present in the pulmonary arteries of PAH patients is one of the oldest histopathological findings in PAH [11]. Over the past decades, multiple investigations have yielded important results on how inflammation and dysregulation of the immune system contribute to pulmonary artery remodeling and PAH [11,12,13]. Yet, immune dysregulation can also exert fundamental effects on endothelial function and contribute to PAH pathophysiology on multiple levels [14]. One less well investigated area is how inflammation and epigenetic changes affect EC function. Here, we are summarizing existing literature to develop a conceptual framework for the regulation of endothelial function via immune dysregulation and epigenetic changes in PAH.

## 2. Endothelial Dysfunction

The endothelium serves many homeostatic functions in the blood vessels including regulation of leukocyte trafficking, gas exchange, and regulation of vascular smooth muscle cell (SMC) function [15,16,17]. It is therefore not surprising that changes in endothelial function have a fundamental effect on many key elements of vascular and immunological biology. Because of the integral role of EC dysfunction in PAH, a variety of scientific and clinical tools as well as assays have been developed to measure endothelial function. In vitro, these assays include the ones that measure the proliferative potential, including clonogenic expansion potential endothelial cell colony forming cell (ECFC) assay and various proliferation assays including the 5-bromodeoxyuridine incorporation assay [18,19]. Additional assays provide an analysis of the migratory and proliferative capacity, including gap closure assays (also known as “wound repair” assays) and angiogenesis assays. Multiple forms of angiogenesis assays have been developed, starting from simple 2-dimensional (2D) network/tube formation assays on a layer of extracellular matrix (ECM) [20]. These assays have been further developed to provide a 3-dimensional, more realistic environment for tube formation, including a 3D sandwich assay or a spheroid sprouting assay [18].

One important determinant of endothelial function plays a prominent role in studying the microvascular capillary endothelium of the lung, particularly in the context of acute lung injury [21]. Lung microvascular ECs have a crucial role in regulating fluid and protein homeostasis of the blood during passage of the lung capillary network [22]. Common during acute lung injury, dysfunctional capillary ECs can allow massive fluid and protein extravasation with detrimental consequences for gas exchange [21]. This dysfunctional aspect of the endothelium is commonly studied by barrier function assays [22]. Some of these assays use the fact that an intact endothelial barrier has a higher electrical impedance than leaky, dysfunctional endothelium, whereas other assays make use of the impaired barrier for plasma proteins, such as albumin, in dysfunctional ECs [23,24]. 

A very important aspect of dysfunctional ECs is the impaired interaction with other mural/vascular cells, particularly vascular smooth muscle cells (SMCs) [25]. ECs produce a variety of mediators that limit vasoconstriction and SMC growth, including nitric oxide, prostacyclin, and bone morphogenetic protein ligands [26,27,28]. Dysfunctional endothelial to SMC crosstalk is derived from the lack or impaired signaling of these EC-derived mediators. The production of mediators that promote vasoconstriction and SMC growth such as endothelin-1 or transforming growth factor-β [29,30,31]. Together, vasodilators and vasoconstrictors function in counterpart of each other to regulate vascular integrity and function. Many potential mechanisms have been investigated over the years that have the potential to initiate and drive endothelial dysfunction in PAH, including genetics and inflammation, but the role of epigenetic regulation of endothelial function has only recently been studied in more detail.

## 3. Epigenetic Regulation of Endothelial Function

Epigenetic regulation links changes in chromatin structure to activation or repression of signaling pathways that alter EC function [32]. Vascular ECs require specific genes in the chromatin to be activated or silenced to maintain homeostasis. These modifications are reversible and impact endothelial gene expression rather than the DNA sequence itself. Increasing evidence has shown that endothelial activation is regulated by epigenetic mechanisms such as DNA methylation, histone modifications, and non-coding RNAs [33,34,35]. Defects in these pathways can lead to increased expression of pro-inflammatory cytokines such as interleukin-6 (IL-6) and tumor necrosis factor- α (TNF-α), nuclear factor kappa B (NF-κB), type I interferons (IFN), and interferon regulatory factors (IRFs), including IRF1 and IRF3. Understanding the wide range of epigenetic mechanisms may help identify potential biomarkers and therapeutic targets for PAH [36,37]. Methods such as chromatin immunoprecipitation (Ch-IP) and RNA-sequencing (RNA-seq) have shed light on pathways that alter endothelial function. Ch-IP assays are primarily used to identify specific genes associated with histone modifying marks whereas RNA-seq can quantitate the expression of spliced variants and non-coding RNAs [38]. Other approaches may include whole-genome reporters to depict a large set of genes regulated by epigenetic change [39].

DNA methylation is a covalent modification that adds a methyl group to the 5-position of cytosine in a sequence, thus yielding a 5-methylcytosine [40]. These modified groups are located within CpG dinucleotides and serve in either recruiting transcriptional repressors, i.e., methyl-CpG binding proteins, or inhibiting the transcription factors at the promoter region, i.e., CCCTC-binding factors (CTCF) [41]. DNA methylation reactions are catalyzed by two types of DNA methyltransferases (DNMTs), DNMT1 and DNMT3A/DNMT3B [42,43,44]. DNMT1 is responsible for maintaining the methylation in the newly synthesized strand and binds to hemi-methylated cytosines [44]. In contrast, DNMT3A and DNMT3B function in *de novo* methylation which does not require hemi-methylation [43,45]. Previous studies highlight the significance of DNA methylation in EC function as well as endothelium-specific expression of endothelial NO synthase (eNOS) via VEGF signaling pathways [33,46]. As the name indicates, the eNOS enzyme serves a critical role in producing nitric oxide in the vascular endothelium which enhances vasodilation and maintains endothelial homeostasis [47]. Furthermore, DNA methylation of the eNOS proximal promoter negatively impacts functional activity as well as vascular tone and angiogenesis. These studies are the first to show regulatory roles for DNA methylation in the constitutively expressed eNOS gene in the vascular endothelium [33]. In addition, DNA methylation activates or inhibits inflammatory pathways associated with PAH [48,49,50].

Another layer of chromatin remodeling is the post-translation modification (PTM) of histones which have been well studied in vascular biology [51]. Gene expression begins with tightly packaged DNA, or chromosomes, that surround histone proteins to form an octamer complex known as a nucleosome [52]. Nucleosomes consists of two histone copies of histones H2A, H2B, H3, and H4 that is further compacted by linker DNA [53]. PTMs modulate the structural changes within the nucleosome and produce histone variants that regulate the transcription of genes. The most notable histone modifications are methylation and acetylation which both regulate inflammation as well as EC function [34]. ECs have distinct modifications of histones at the core promoter, specifically, enriched di- and tri-methylation of histone H3 lysine 4 (H3K4) and acetylation of histone H3 lysine 9 (H3K9), and histone H4 lysine 12 (H4K12) [34]. Histone methyltransferases (HMTs) are enzymes that regulate transcription by transferring one, two, or three methyl groups to the histones [54]. Some of the most prominent HMTs involved in endothelial gene expression are EZH2 and ASH2 [55]. Histone modifications such as H3K27me3 are associated with gene silencing, or inactivation, and contribute to altered endothelial gene expression [56,57]. Another histone modification, histone acetylation, activates gene transcription by adding negatively charged acetyl groups to DNA, ultimately relaxing the chromatin [58]. Acetylation of histones is performed by histone acetyltransferase (HATs) enzymes that transfer acetyl groups to conserved lysine residues and are linked to transcriptional activation [59]. Specific HATs, such as histone acetyltransferase 7 (KAT7) modulates endothelial function by activating the transcription of vascular endothelial growth factors (VEGFs) [60]. It was shown that KAT7 is necessary for VEGFR-2 transcription and blood vessel formation in zebrafish embryos [60]. 

Despite the lack of studies on RNA-based mechanisms, non-coding RNAs are recognized for their regulation of post-transcriptional activities in ECs. While non-coding RNAs are not epigenetic per se, their expression is frequently regulated by epigenetic modification of their respective gene promoter [61,62,63,64,65]. Non-coding RNAs such as microRNA (miRNA) and long coding RNA (lncRNA) can dysregulate EC function by permitting smooth muscle cell proliferation and EnMT [66,67]. Long non-coding RNAs are defined as being larger than 200 nucleotides whereas the average size of miRNAs is approximately 20–21 nucleotides in length [68,69]. Microarray analysis has helped construct a spheroid model of miRNA, including miR-424-5p and miR-29a-3p that promote endothelial migration and repress genes in the VEGF pathway [70]. These studies have also validated the regulatory roles of miRNAs in the Mitogen-Associated Protein Kinase (MAPK) pathway which mediates VEGF-A-induced cell proliferation [70]. Other studies have shown the regulation of vascular remodeling and angiogenesis through the combined functions of novel miRNAs such as miR-181a-5p and miR-324-5p [71]. The investigation of long non-coding RNAs is also an emerging field in endothelial dysfunction and vascular biology [72]. Past studies have used chromosome- and genome-wide screening of cDNA libraries to show high expression of lncRNAs in the human genome [73]. Among the novel lncRNAs expressed in ECs are metastasis associated lung adenocarcinoma transcript 1 (MALAT1), a regulator of glucose-induced production by the inflammatory cytokines IL-6 and TNF-α [74]. Studies show that MALAT1 is increased in hypoxia condition and the knockdown of the lncRNA decreases proliferation and regulates endothelial functions, such as angiogenesis [75]. Other lncRNAs such as antisense non-coding RNA in the INK4 locus (ANRIL) and non-coding RNA activated by DNA damage (NORAD) also causes endothelial dysfunction by impeding apoptosis and increasing proliferation [76,77]. 

Because both ECs and smooth muscle cells (SMCs) from PAH patients maintain features of dysfunction In vitro, it has been widely assumed that epigenetic modifications occur in multiple cell types of PAH patients which also contributes to pathogenesis and disease progression [78,79,80,81]. These studies are not limited to human patients as others have used newborn rat models to investigate the epigenetic regulation of eNOS expression [82]. Up to date, more epigenetic changes have been identified in pulmonary artery SMCs than in ECs [51,62,83,84,85,86,87]. Some studies suggest a potential role for SMC contact and endothelial metabolism in chromatin remodeling leading to EC regeneration, however, this level of gene regulation remains unclear [88]. Epigenetic modifications also modulate EnMT, lung vascular and right ventricular remodeling, as well as novel transcriptional targets for idiopathic PAH [89,90,91]. Important epigenetic mechanisms that have been identified in PAH include promoter DNA hypermethylation in pulmonary ECs from PAH patients that particularly affected the expression of genes within the lipid metabolism [62]. Further, in a hypoxic fetal lamb model of pulmonary hypertension (PH), expression of eNOS was decreased, and this reduction was associated with epigenetic changes, including promoter hypermethylation and histone acetylation and trimethylation [87]. A novel mechanism of endothelial dysfunction in PAH was based on reduced expression of BolA Family Member 3 (BOLA3) and its transcription was inhibited via histone deacetylation [92]. Extensive remodeling of H3K27AC may leads to aberrant gene-regulatory networks in PAECs in the context of PAH [93]. A combination of altered transcription factor activity and remodeling of active endothelial enhancers was found using prediction analysis in PAH ECs [93]. We have summarized the important studies regarding epigenetic regulation of endothelial function and PAH in Table 1.

Besides epigenetic changes, inflammation has a key role in the development and progression of PAH and to understand the role of inflammation in epigenetic regulation, it is imperative to immerse further into the detailed molecular mechanisms that drive inflammation and immune dysregulation [11,13].

## 4. PAMPs and DAMPs

ECs serve as the primary defensive response against microbial infection and tissue injury in the vascular system. An important function of ECs is to recognize stress-associated signals that activate an inflammatory response leading to defects in endothelial structure and function. Pathogen-associated molecular pattern (PAMPs) and damage-associated molecular patterns (DAMPs) are two classes of ligands that are detected by host cells through a family of pattern recognition receptors (PRRs) [95]. PAMPs are exogenic molecules produced by microorganisms which includes lipoteichoic acids (LTAs), lipopolysaccharides (LPSs), peptidoglycan, flagellin, lipoproteins, and double stranded (ds) RNA [96]. In contrast, DAMPs are endogenous molecules secreted from damaged or dying cells that are recognized by the same PRRs as PAMPs and thereby activate similar pathways as PAMPs [97]. Notable DAMPs are DNA released from replication stress, heat shock proteins, S100 proteins, and high mobility group box 1 (HMGB-1). Also known as signal 0s, PAMPs and DAMPs bind to highly conserved regions on PRRs and stimulate intracellular signaling [98]. This interaction stimulates a signaling cascade responsible for upregulating the expression of type I IFNs, pro-inflammatory cytokines, chemokines, and other mediators that can promote endothelial dysfunction in the pulmonary circulation [11,12,15]. As a result, ECs undergo apoptosis or the more specialized pyro-apoptosis, and exhibit impaired barrier function, causing decreased blood flow [67,76,99,100,101,102,103,104].

Past research has focused on how PAMPs and DAMPs activate specific PRR-mediated inflammatory response pathways amplifying cardiovascular disease [97,105]. PAMP/DAMPs also induce inflammatory pathways in response to severe acute respiratory syndrome (SARS)-coronavirus (CoV) and Middle East respiratory syndrome CoV (MERS) infections [103,106]. RNA sequencing of virus-infected ECs yields upregulation of innate immune regulator genes (IGs) in response to PAMPS/DAMPS such as lipopolysaccharide (LPS) and oxidized low density lipoproteins (oxLDL) [103]. IG induction result in transcriptomic changes leading to thrombosis specifically in microvascular ECs [103].

There are several families of PRRs that recognize PAMPs and DAMPs including toll-like receptors (TLRs), C-type lectin receptors (CLRs), retinoic acid-inducible gene (RIG)-I-like receptors (RLRs), NOD-like receptors (NLRs), and leucin rich repeats-containing receptors (LRRs) [98,107,108,109]. TLRs are highly conserved type I membrane glycoproteins that were first discovered in *Drosophila melanogaster* [110,111]. Humans and mice have 10 and 12 known TLRs, respectively [105,109]. TLRs either exist as preformed dimers or form homo- or heterodimers following binding of PAMPs and DAMPs, subsequently leading to the activation of signaling pathways mediated by NF-κB, IRFs, and MAP kinases [112]. As a result, TLR stimulation causes EC activation, cell proliferation, inflammation, and ultimately PAH [111]. TLR activation and inflammation can lead to the development of PAH through several mechanisms. One of which involves the increased expression of endothelin-1(ET-1), an endogenous vasoconstrictor released from the vascular smooth muscle of pulmonary arteries [113,114].

The subcellular localization of TLRs is tightly controlled at regions such as the endosomes and lysosomes [18,115]. TLRs located at the cell surface, such as TLR1, 2, 4, 5, 6, and 11, bind lipids, lipoproteins, and other membrane molecules [96,106,110,116]. In contrast, the TLRs 3, 7, 8, and 9 are located at membranes of intracellular vesicles, such as endosomes [96,106,110,113,116]. TLR2 and TLR4 signaling is triggered by PAMP molecules such as LPS and oxidized phospholipids. TLR3 is a nucleotide-sensor of dsRNA, which is a result of viral pathogens, and other self-RNAs including mRNA [112,116]. Further, TLR3 affects multiple fundamental aspects of cellular function, including autophagy and apoptosis [117,118]. In the systemic and the pulmonary circulation, TLR3 has a protective function, and our group has shown that silencing of TLR3 expression in pulmonary artery ECs promotes apoptosis, whereas a TLR3 agonist reduced PH in rats [119,120]. However, other reports also demonstrate that severe systemic arterial injury predisposes to increased vascular injury following activation of TLR3 [121]. In models of PH, various TLR4 ligands, including high mobility group box 1 promote PH in experimental models and genetic knockout of TLR4 prevents PH in mice [122,123,124,125,126,127,128,129]. Our group has shown that endothelial TLR3 deficiency contributes to PAH via increased endothelial apoptosis [120]. In addition, treatment with a TLR3 agonist reverses severe PH in a rodent model [120]. To further understand the effects of PRR signaling, a closer look needs to be taken into central pro-inflammatory pathways, which are frequently activated downstream of PRRs.

## 5. Central Pathways Driving Inflammation

Endothelial dysfunction is driven by pro-inflammatory mediators that act downstream of PRR signaling pathways [1,101]. These pathways specifically induce genes encoding cytokines, chemokines, IRF transcription factors, adhesion molecules, and regulators that recruit leukocytes to sites of damage and infection. Upon recognizing PAMPs and DAMPs, PRRs, such as TLRs, induce an intracellular signaling cascade defined by several phosphorylation events as PTMs [106,110,116]. The combined efforts of pro-inflammatory factors affect inflammation, coagulation, endothelial barrier permeability, and gas exchange between the blood and lung tissue [11]. Although inflammatory response factors help protect against additional damage to the endothelium, these pathways lead to pathogenesis of PAH [11].

TLR signaling, for example, primarily induces a type I IFN response leading to the transcription of specific cytokines, such as IL-6, CXC chemokine ligand 8 (CXCL8), and CC chemokine ligand 2 (CCL2) as well as other stimulatory molecules [96,106,110,116]. TLR activation can also lead to activation of ECs which is controlled by gene expression [130]. These cytokines also help promote angiogenesis, recruit leukocytes, and upregulate leukocyte adhesion molecules in ECs [131,132,133]. Vascular ECs have contrasting classic- and trans-signaling of IL-6 [134]. Demethylation in the promoter activates transcription of the IL-6 gene in ECs [135], a mechanism that could also play a role in PAH. The following paragraphs discuss potential disease specific mechanisms in a selected set of pathways.

Nuclear factor-κB (NF-κB) is a central switchboard of inflammation in most cell types of the body [136]. Multiple inflammatory signals, including downstream of TNF-α or TLRs, induce NF-κB activation. The canonical NF-κΒ pathway requires nuclear translocation of a p65/p50 dimer to the nucleus, leading to activation of gene transcription [136]. Activation of this pathway is a key player in vascular and cardiac remodeling, including in PAH and experimental models of severe PH, as shown by our group [94,137,138,139,140,141,142,143]. Our group has also shown that inhibition of NF-κΒ reduces occlusive pulmonary arteriopathy in severe PH in rats [141]. Epigenetic modifications, including histone deacetylation, miRNAs or demethylation, can alter NF-κΒ activity and hence may contribute to the amplification of NF-κB signaling in pulmonary arteries from PAH patients [144,145,146,147,148,149].

Similar to NF-κB, IRF3 and IRF7 are key transcription factors downstream of many PRRs, including TLR3 and TLR4 [150,151,152,153]. Phosphorylation and nuclear translocation of IRF3 and IRF7 promote transcription of type I IFNs IFN-α and IFN-β, which triggers a succinct signaling cascade with strong antiviral activity [150,151,152,153,154,155]. However, the role of type I IFNs is controversial in PAH. On one side, patients with significant pre-existing morbidity, i.e., Multiple Sclerosis, can develop a reversible form of PH under IFN therapy [156,157,158]. A potential adverse role for type I IFN has been suggested by the protective effect of type I IFN receptor knockout for chronic hypoxic PH in mice [12]. However, type I IFN treatment decreased severe PH in rats, suggesting a context- and disease phenotype-specific role for interferons in lung vascular remodeling [159]. Some of the differences could be explained by epigenetic modification of IRF3 and IRF7 signaling molecules, such as their methylation status, which can alter the extent of downstream signaling [160,161]. Yet, more work is necessary to understand this interaction in ECs.

Hypoxia is a well-known trigger for lung vascular remodeling and endothelial dysfunction [162,163,164,165,166,167]. Hypoxia signaling is mediated via the hypoxia-inducible factors (HIFs), which are stabilized in the cell following reduced oxygen levels and induce gene transcription by activation of hypoxia-responsive elements (HRE) in the promoter of target genes [168]. Three isoforms of HIF have been shown in humans, HIF-1α, HIF-2^α^, and HIF-3α that show a vast variety in cell-specific functions [169]. Under normal conditions HIF-1α, and HIF-2α are rapidly degraded, however, during hypoxia the degradation of HIF-1α and HIF-2α is inhibited, hence contributing to the development of PAH [170,171]. HIF-1α is considered the master regulator of homestatic response to hypoxia by promoting the transcription of genes involved in angiogenesis, vasculogenesis, apoptosis, and energy metabolism [172,173,174]. Important targets of HIF-1α are vascular endothelial growth factors (VEGFs) which bind to and stimulate pro-angiogenic receptors as well as promote vascular permeability [175,176]. In contrast, HIF-2α has a distinct role in promoting EnMT, which contributes to pulmonary vascular remodeling [171]. In addition, HIF1α expression is rapidly increased following induced hypoxia whereas HIF-2α is known to gradually accumulate later in time [170].There is a variety of ways by which HIF transcription factors affect inflammation and epigenetics, including in ECs. One example is activation of NF-κΒ signaling by hypoxia via the prolyl hydroxylases that also regulate HIF activity and degradation [169]. Hypoxia and HIFs have been shown to induce significant functional changes in ECs in PH, including metabolic reprogramming in ECs, dysfunction of endothelial colony-forming cells, impaired angiogenesis, and altered estrogen metabolism via HIF-1α [177,178,179,180]. More recently, HIF-2α has been identified as another major driver of lung vascular remodeling and PH, and HIF-2α drives EnMT, the process by which ECs change phenotype and function to a myofibroblast/SMC-like cell [171,181,182,183]. EnMT promotes lung vascular remodeling and contributes to the dysfunctional endothelial phenotype in PAH [9,184,185]. Interestingly, EnMT is regulated via epigenetic modification of multiple potential target genes [62,93]. In addition, hypoxia and HIFs can induce various epigenetic changes, including histone acetylation and methylation [61,64,94,186,187].

## 6. Conclusions

Epigenetic modifications and inflammation have significant effects on the function of the pulmonary artery endothelium, and thereby contribute to the pathogenesis of PAH. While epigenetics and inflammation also contribute to PAH by affecting cellular signaling in other important cell types and organs, including pulmonary artery SMCs, pulmonary artery adventitia fibroblasts and the right ventricle [61,85,188,189]. Although the complete mechanisms of epigenetic regulation of endothelial dysfunction remain unclear, here we summarize the current literature on putative modifications and pathways leading to inflammation, lung vascular remodeling and PAH. The development of novel therapies targeting these changes will open new possibilities to treat this life-threatening disease.

## Figures and Tables

**Table 1:** Previous literature of putative epigenetic pathways and regulatory factors associated with PAH and endothelial dysfunction.

	Source	Epigenetic Regulators	Primary Model	Description:
DNA methylation	Chan et al. (2004) [33]	multiple	mice	eNOS expression
	Fish et al. (2005) [34]	multiple	in vitro	eNOS expression
	Quentmeier et al. (2012) [46]	multiple	in vitro	angiogenesis
	Hautefort et al. (2017) [62]	multiple	in vitro	proliferation; EnMT, inflammation
	Wang et al. (2018) [49]	N/A	patients	vascular remodeling
	Ke et al. (2018) [87]	Specificity Protein 1	in vitro/lamb	eNOS expression
	Yan et al. (2020) [45]	DNMT3B	rat	vascular remodeling
	Joshi et al. (2020) [48]	multiple	mice	mitochondrial metabolism
Histone modifications	Maleszewska et al. (2016) [55]	EZH2	in vitro	EC expression
	Hulshoff et al. (2018) [89]	multiple	in vitro	EnMT
	Li et al. (2018) [94]	JARID1B	in vitro	proliferation
	Yan et al. (2018) [60]	KAT7	zebrafish	angiogenesis
	Yu et al. (2019) [92]	BOLA3	in vitro	EC metabolism
Non-coding RNAs	Michalik et al. (2014) [75]	MALAT1	in vitro	proliferation
	Simion et al. (2019) [72]	multiple	in vitro	angiogenesis
	Puthanveetil et al. (2015) [74]	MALAT1	in vitro/mice	inflammation
	Rosano et al. (2020) [70]	miR-424-5p, miR-29a-3p	in vitro	EC expression
	Sindi et al. (2020) [71]	miR-181a-5p, miR-324-5p	in vitro	proliferation; angiogenesis
	Liu et al. (2020) [76]	ANRIL	patients	EC dysfunction
	Bian et al. (2021) [77]	NORAD	in vitro	proliferation; EnMT

## Data Availability

Not applicable.

## References

[1] Evans C.E., Cober N.D., Dai Z., Stewart D.J., Zhao Y.Y. (2021). Endothelial cells in the pathogenesis of pulmonary arterial hypertension. Eur. Respir. J..

[2] Jensen-Urstad K., Johansson J., Jensen-Urstad M. (1997). Vascular function correlates with risk factors for cardiovascular disease in a healthy population of 35-year-old subjects. J. Intern. Med..

[3] Incalza M.A., D’Oria R., Natalicchio A., Perrini S., Laviola L., Giorgino F. (2018). Oxidative stress and reactive oxygen species in endothelial dysfunction associated with cardiovascular and metabolic diseases. Vascul. Pharmacol..

[4] Niemann B., Rohrbach S., Miller M.R., Newby D.E., Fuster V., Kovacic J.C. (2017). Oxidative Stress and Cardiovascular Risk: Obesity, Diabetes, Smoking, and Pollution: Part 3 of a 3-Part Series. J. Am. Coll. Cardiol..

[5] Denekamp J., Hobson B. (1982). Endothelial-cell proliferation in experimental tumours. Br. J. Cancer.

[6] Yu Q., Chan S.Y. (2017). Mitochondrial and Metabolic Drivers of Pulmonary Vascular Endothelial Dysfunction in Pulmonary Hypertension. Adv. Exp. Med. Biol..

[7] Ashford T.P., Frieiman D.G. (1967). The role of the endothelium in the initial phases of thrombosis. An electron microscopic study. Am. J. Pathol..

[8] Becker B.F., Heindl B., Kupatt C., Zahler S. (2000). Endothelial function and hemostasis. Z. Kardiol..

[9] Good R.B., Gilbane A.J., Trinder S.L., Denton C.P., Coghlan G., Abraham D.J., Holmes A.M. (2015). Endothelial to Mesenchymal Transition Contributes to Endothelial Dysfunction in Pulmonary Arterial Hypertension. Am. J. Pathol..

[10] Conway E.M., Carmeliet P. (2004). The diversity of endothelial cells: A challenge for therapeutic angiogenesis. Genome Biol..

[11] Rabinovitch M., Guignabert C., Humbert M., Nicolls M.R. (2014). Inflammation and immunity in the pathogenesis of pulmonary arterial hypertension. Circ. Res..

[12] George P.M., Oliver E., Dorfmuller P., Dubois O.D., Reed D.M., Kirkby N.S., Mohamed N.A., Perros F., Antigny F., Fadel E. (2014). Evidence for the involvement of type I interferon in pulmonary arterial hypertension. Circ. Res..

[13] Stacher E., Graham B.B., Hunt J.M., Gandjeva A., Groshong S.D., McLaughlin V.V., Jessup M., Grizzle W.E., Aldred M.A., Cool C.D. (2012). Modern age pathology of pulmonary arterial hypertension. Am. J. Respir. Crit. Care Med..

[14] Thenappan T., Ormiston M.L., Ryan J.J., Archer S.L. (2018). Pulmonary arterial hypertension: Pathogenesis and clinical management. BMJ.

[15] Bevilacqua M.P., Pober J.S., Wheeler M.E., Cotran R.S., Gimbrone M.A. (1985). Interleukin-1 activation of vascular endothelium. Effects on procoagulant activity and leukocyte adhesion. Am. J. Pathol..

[16] Helewski K., Konecki J., Kowalczyk G. (1989). Structural aspects of pulmonary gas exchange. II. Alveolar epithelium. Endothelium of capillaries of the alveolar wall. Pneumonol. Pol..

[17] Rapoport R.M., Murad F. (1983). Endothelium-dependent and nitrovasodilator-induced relaxation of vascular smooth muscle: Role of cyclic GMP. J. Cyclic Nucleotide Protein Phosphor. Res..

[18] Bhagwani A.R., Farkas D., Harmon B., Authelet K.J., Cool C.D., Kolb M., Goncharova E., Yoder M.C., Clauss M., Freishtat R. (2020). Clonally selected primitive endothelial cells promote occlusive pulmonary arteriopathy and severe pulmonary hypertension in rats exposed to chronic hypoxia. Sci. Rep..

[19] Ingram D.A., Mead L.E., Tanaka H., Meade V., Fenoglio A., Mortell K., Pollok K., Ferkowicz M.J., Gilley D., Yoder M.C. (2004). Identification of a novel hierarchy of endothelial progenitor cells using human peripheral and umbilical cord blood. Blood.

[20] Ingram D.A., Mead L.E., Moore D.B., Woodard W., Fenoglio A., Yoder M.C. (2005). Vessel wall-derived endothelial cells rapidly proliferate because they contain a complete hierarchy of endothelial progenitor cells. Blood.

[21] Ince C., Mayeux P.R., Nguyen T., Gomez H., Kellum J.A., Ospina-Tascón G.A., Hernandez G., Murray P., De Backer D. (2016). The Endothelium in Sepsis. Shock.

[22] Adil M.S., Somanath P.R. (2021). Endothelial Permeability Assays In Vitro. Methods Mol. Biol..

[23] Saunders N.R., Dziegielewska K.M., Møllgård K., Habgood M.D. (2015). Markers for blood-brain barrier integrity: How appropriate is Evans blue in the twenty-first century and what are the alternatives?. Front. Neurosci..

[24] Sun T., Swindle E.J., Collins J.E., Holloway J.A., Davies D.E., Morgan H. (2010). On-chip epithelial barrier function assays using electrical impedance spectroscopy. Lab Chip.

[25] Humbert M., Montani D., Perros F., Dorfmüller P., Adnot S., Eddahibi S. (2008). Endothelial cell dysfunction and cross talk between endothelium and smooth muscle cells in pulmonary arterial hypertension. Vascul. Pharmacol..

[26] Palmer R.M., Ashton D.S., Moncada S. (1988). Vascular endothelial cells synthesize nitric oxide from L-arginine. Nature.

[27] Weksler B.B., Marcus A.J., Jaffe E.A. (1977). Synthesis of prostaglandin I2 (prostacyclin) by cultured human and bovine endothelial cells. Proc. Natl. Acad. Sci. USA.

[28] Wong C.M., Zhang Y., Huang Y. (2014). Bone morphogenic protein-4-induced oxidant signaling via protein carbonylation for endothelial dysfunction. Free Radic. Biol. Med..

[29] Fràter-Schröder M., Müller G., Birchmeier W., Böhlen P. (1986). Transforming growth factor-beta inhibits endothelial cell proliferation. Biochem. Biophys. Res. Commun..

[30] Horgan M.J., Pinheiro J.M., Malik A.B. (1991). Mechanism of endothelin-1-induced pulmonary vasoconstriction. Circ. Res..

[31] Makino A., Firth A.L., Yuan J.X. (2011). Endothelial and smooth muscle cell ion channels in pulmonary vasoconstriction and vascular remodeling. Compr. Physiol..

[32] Chan G.C., Fish J.E., Mawji I.A., Leung D.D., Rachlis A.C., Marsden P.A. (2005). Epigenetic basis for the transcriptional hyporesponsiveness of the human inducible nitric oxide synthase gene in vascular endothelial cells. J. Immunol..

[33] Chan Y., Fish J.E., D’Abreo C., Lin S., Robb G.B., Teichert A.M., Karantzoulis-Fegaras F., Keightley A., Steer B.M., Marsden P.A. (2004). The cell-specific expression of endothelial nitric-oxide synthase: A role for DNA methylation. J. Biol. Chem..

[34] Fish J.E., Matouk C.C., Rachlis A., Lin S., Tai S.C., D’Abreo C., Marsden P.A. (2005). The expression of endothelial nitric-oxide synthase is controlled by a cell-specific histone code. J. Biol. Chem..

[35] Qin B., Yang H., Xiao B. (2012). Role of microRNAs in endothelial inflammation and senescence. Mol. Biol. Rep..

[36] Pullamsetti S.S., Perros F., Chelladurai P., Yuan J., Stenmark K. (2016). Transcription factors, transcriptional coregulators, and epigenetic modulation in the control of pulmonary vascular cell phenotype: Therapeutic implications for pulmonary hypertension (2015 Grover Conference series). Pulm. Circ..

[37] Xu X.F., Cheng F., Du L.Z. (2011). Epigenetic regulation of pulmonary arterial hypertension. Hypertens. Res..

[38] Mimura I., Kanki Y., Kodama T., Nangaku M. (2014). Revolution of nephrology research by deep sequencing: ChIP-seq and RNA-seq. Kidney Int..

[39] Cazaly E., Saad J., Wang W., Heckman C., Ollikainen M., Tang J. (2019). Making Sense of the Epigenome Using Data Integration Approaches. Front. Pharmacol..

[40] Walsh C.P., Chaillet J.R., Bestor T.H. (1998). Transcription of IAP endogenous retroviruses is constrained by cytosine methylation. Nat. Genet..

[41] Bird A.P. (1986). CpG-rich islands and the function of DNA methylation. Nature.

[42] Leonhardt H., Bestor T.H. (1993). Structure, function and regulation of mammalian DNA methyltransferase. EXS.

[43] Okano M., Bell D.W., Haber D.A., Li E. (1999). DNA methyltransferases Dnmt3a and Dnmt3b are essential for de novo methylation and mammalian development. Cell.

[44] Yoder J.A., Soman N.S., Verdine G.L., Bestor T.H. (1997). DNA (cytosine-5)-methyltransferases in mouse cells and tissues. Studies with a mechanism-based probe. J. Mol. Biol..

[45] Yan Y., He Y.Y., Jiang X., Wang Y., Chen J.W., Zhao J.H., Ye J., Lian T.Y., Zhang X., Zhang R.J. (2020). DNA methyltransferase 3B deficiency unveils a new pathological mechanism of pulmonary hypertension. Sci. Adv..

[46] Quentmeier H., Eberth S., Romani J., Weich H.A., Zaborski M., Drexler H.G. (2012). DNA methylation regulates expression of VEGF-R2 (KDR) and VEGF-R3 (FLT4). BMC Cancer.

[47] Giraldez R.R., Panda A., Xia Y., Sanders S.P., Zweier J.L. (1997). Decreased nitric-oxide synthase activity causes impaired endothelium-dependent relaxation in the postischemic heart. J. Biol. Chem..

[48] Joshi S.R., Kitagawa A., Jacob C., Hashimoto R., Dhagia V., Ramesh A., Zheng C., Zhang H., Jordan A., Waddell I. (2020). Hypoxic activation of glucose-6-phosphate dehydrogenase controls the expression of genes involved in the pathogenesis of pulmonary hypertension through the regulation of DNA methylation. Am. J. Physiol. Lung Cell. Mol. Physiol..

[49] Wang Y., Huang X., Leng D., Li J., Wang L., Liang Y., Wang J., Miao R., Jiang T. (2018). DNA methylation signatures of pulmonary arterial smooth muscle cells in chronic thromboembolic pulmonary hypertension. Physiol. Genom..

[50] Xing X.Q., Li B., Xu S.L., Zhang C.F., Liu J., Deng Y.S., Yang J. (2019). 5-Aza-2′-deoxycytidine, a DNA methylation inhibitor, attenuates hypoxic pulmonary hypertension via demethylation of the PTEN promoter. Eur. J. Pharmacol..

[51] Chelladurai P., Boucherat O., Stenmark K., Kracht M., Seeger W., Bauer U.M., Bonnet S., Pullamsetti S.S. (2021). Targeting histone acetylation in pulmonary hypertension and right ventricular hypertrophy. Br. J. Pharmacol..

[52] Oudet P., Germond J.E., Bellard M., Spadafora C., Chambon P. (1978). Nucleosome structure. Philos. Trans. R Soc. Lond. B Biol. Sci..

[53] Luger K., Mäder A.W., Richmond R.K., Sargent D.F., Richmond T.J. (1997). Crystal structure of the nucleosome core particle at 2.8 A resolution. Nature.

[54] Kouzarides T. (2002). Histone methylation in transcriptional control. Curr. Opin. Genet. Dev..

[55] Maleszewska M., Vanchin B., Harmsen M.C., Krenning G. (2016). The decrease in histone methyltransferase EZH2 in response to fluid shear stress alters endothelial gene expression and promotes quiescence. Angiogenesis.

[56] Cao R., Wang L., Wang H., Xia L., Erdjument-Bromage H., Tempst P., Jones R.S., Zhang Y. (2002). Role of histone H3 lysine 27 methylation in Polycomb-group silencing. Science.

[57] Xu S., Xu Y., Yin M., Zhang S., Liu P., Koroleva M., Si S., Little P.J., Pelisek J., Jin Z.G. (2018). Flow-dependent epigenetic regulation of IGFBP5 expression by H3K27me3 contributes to endothelial anti-inflammatory effects. Theranostics.

[58] Grunstein M. (1997). Histone acetylation in chromatin structure and transcription. Nature.

[59] Davie J.R., Hendzel M.J. (1994). Multiple functions of dynamic histone acetylation. J. Cell Biochem..

[60] Yan M.S., Turgeon P.J., Man H.J., Dubinsky M.K., Ho J.J.D., El-Rass S., Wang Y.D., Wen X.Y., Marsden P.A. (2018). Histone acetyltransferase 7 (KAT7)-dependent intragenic histone acetylation regulates endothelial cell gene regulation. J. Biol. Chem..

[61] Chouvarine P., Legchenko E., Geldner J., Riehle C., Hansmann G. (2019). Hypoxia drives cardiac miRNAs and inflammation in the right and left ventricle. J. Mol. Med..

[62] Hautefort A., Chesné J., Preussner J., Pullamsetti S.S., Tost J., Looso M., Antigny F., Girerd B., Riou M., Eddahibi S. (2017). Pulmonary endothelial cell DNA methylation signature in pulmonary arterial hypertension. Oncotarget.

[63] Li Y., Li L., Qian Z., Lin B., Chen J., Luo Y., Qu J., Raj J.U., Gou D. (2018). Phosphatidylinositol 3-Kinase-DNA Methyltransferase 1-miR-1281-Histone Deacetylase 4 Regulatory Axis Mediates Platelet-Derived Growth Factor-Induced Proliferation and Migration of Pulmonary Artery Smooth Muscle Cells. J. Am. Heart Assoc..

[64] Nallamshetty S., Chan S.Y., Loscalzo J. (2013). Hypoxia: A master regulator of microRNA biogenesis and activity. Free Radic. Biol. Med..

[65] Zhang H., Laux A., Stenmark K.R., Hu C.J. (2021). Mechanisms Contributing to the Dysregulation of miRNA-124 in Pulmonary Hypertension. Int. J. Mol. Sci..

[66] Hulshoff M.S., Del Monte-Nieto G., Kovacic J., Krenning G. (2019). Non-coding RNA in endothelial-to-mesenchymal transition. Cardiovasc. Res..

[67] Shan K., Jiang Q., Wang X.Q., Wang Y.N., Yang H., Yao M.D., Liu C., Li X.M., Yao J., Liu B. (2016). Role of long non-coding RNA-RNCR3 in atherosclerosis-related vascular dysfunction. Cell Death Dis..

[68] Ambros V., Lee R.C., Lavanway A., Williams P.T., Jewell D. (2003). MicroRNAs and other tiny endogenous RNAs in C. elegans. Curr. Biol..

[69] Kurokawa R. (2011). Long noncoding RNA as a regulator for transcription. Prog. Mol. Subcell. Biol..

[70] Rosano S., Corà D., Parab S., Zaffuto S., Isella C., Porporato R., Hoza R.M., Calogero R.A., Riganti C., Bussolino F. (2020). A regulatory microRNA network controls endothelial cell phenotypic switch during sprouting angiogenesis. eLife.

[71] Sindi H.A., Russomanno G., Satta S., Abdul-Salam V.B., Jo K.B., Qazi-Chaudhry B., Ainscough A.J., Szulcek R., Jan Bogaard H., Morgan C.C. (2020). Therapeutic potential of KLF2-induced exosomal microRNAs in pulmonary hypertension. Nat. Commun..

[72] Simion V., Haemmig S., Feinberg M.W. (2019). LncRNAs in vascular biology and disease. Vascul. Pharmacol..

[73] Clark M.B., Johnston R.L., Inostroza-Ponta M., Fox A.H., Fortini E., Moscato P., Dinger M.E., Mattick J.S. (2012). Genome-wide analysis of long noncoding RNA stability. Genome. Res..

[74] Puthanveetil P., Chen S., Feng B., Gautam A., Chakrabarti S. (2015). Long non-coding RNA MALAT1 regulates hyperglycaemia induced inflammatory process in the endothelial cells. J. Cell. Mol. Med..

[75] Michalik K.M., You X., Manavski Y., Doddaballapur A., Zörnig M., Braun T., John D., Ponomareva Y., Chen W., Uchida S. (2014). Long noncoding RNA MALAT1 regulates endothelial cell function and vessel growth. Circ. Res..

[76] Liu X., Li S., Yang Y., Sun Y., Yang Q., Gu N., Li J., Huang T., Liu Y., Dong H. (2021). The lncRNA ANRIL regulates endothelial dysfunction by targeting the let-7b/TGF-βR1 signalling pathway. J. Cell. Physiol..

[77] Bian W., Jing X., Yang Z., Shi Z., Chen R., Xu A., Wang N., Jiang J., Yang C., Zhang D. (2020). Downregulation of LncRNA NORAD promotes Ox-LDL-induced vascular endothelial cell injury and atherosclerosis. Aging.

[78] Masri F.A., Xu W., Comhair S.A., Asosingh K., Koo M., Vasanji A., Drazba J., Anand-Apte B., Erzurum S.C. (2007). Hyperproliferative apoptosis-resistant endothelial cells in idiopathic pulmonary arterial hypertension. Am. J. Physiol. Lung Cell. Mol. Physiol..

[79] Perros F., Sentenac P., Boulate D., Manaud G., Kotsimbos T., Lecerf F., Lamrani L., Fadel E., Mercier O., Londono-Vallejo A. (2019). Smooth Muscle Phenotype in Idiopathic Pulmonary Hypertension: Hyper-Proliferative but not Cancerous. Int. J. Mol. Sci..

[80] Tajsic T., Morrell N.W. (2011). Smooth muscle cell hypertrophy, proliferation, migration and apoptosis in pulmonary hypertension. Compr. Physiol..

[81] Xu W., Koeck T., Lara A.R., Neumann D., DiFilippo F.P., Koo M., Janocha A.J., Masri F.A., Arroliga A.C., Jennings C. (2007). Alterations of cellular bioenergetics in pulmonary artery endothelial cells. Proc. Natl. Acad. Sci. USA.

[82] Xu X.F., Ma X.L., Shen Z., Wu X.L., Cheng F., Du L.Z. (2010). Epigenetic regulation of the endothelial nitric oxide synthase gene in persistent pulmonary hypertension of the newborn rat. J. Hypertens..

[83] Bisserier M., Mathiyalagan P., Zhang S., Elmastour F., Dorfmüller P., Humbert M., David G., Tarzami S., Weber T., Perros F. (2021). Regulation of the Methylation and Expression Levels of the BMPR2 Gene by SIN3a as a Novel Therapeutic Mechanism in Pulmonary Arterial Hypertension. Circulation.

[84] Blissenbach B., Nakas C.T., Krönke M., Geiser T., Merz T.M., Pichler Hefti J. (2018). Hypoxia-induced changes in plasma micro-RNAs correlate with pulmonary artery pressure at high altitude. Am. J. Physiol. Lung Cell. Mol. Physiol..

[85] Chen K.H., Dasgupta A., Lin J., Potus F., Bonnet S., Iremonger J., Fu J., Mewburn J., Wu D., Dunham-Snary K. (2018). Epigenetic Dysregulation of the Dynamin-Related Protein 1 Binding Partners MiD49 and MiD51 Increases Mitotic Mitochondrial Fission and Promotes Pulmonary Arterial Hypertension: Mechanistic and Therapeutic Implications. Circulation.

[86] Chouvarine P., Geldner J., Giagnorio R., Legchenko E., Bertram H., Hansmann G. (2020). Trans-Right-Ventricle and Transpulmonary MicroRNA Gradients in Human Pulmonary Arterial Hypertension. Pediatr. Crit. Care Med..

[87] Ke X., Johnson H., Jing X., Michalkiewicz T., Huang Y.W., Lane R.H., Konduri G.G. (2018). Persistent pulmonary hypertension alters the epigenetic characteristics of endothelial nitric oxide synthase gene in pulmonary artery endothelial cells in a fetal lamb model. Physiol. Genom..

[88] Miyagawa K., Shi M., Chen P.I., Hennigs J.K., Zhao Z., Wang M., Li C.G., Saito T., Taylor S., Sa S. (2019). Smooth Muscle Contact Drives Endothelial Regeneration by BMPR2-Notch1-Mediated Metabolic and Epigenetic Changes. Circ. Res..

[89] Hulshoff M.S., Xu X., Krenning G., Zeisberg E.M. (2018). Epigenetic Regulation of Endothelial-to-Mesenchymal Transition in Chronic Heart Disease. Arterioscler. Thromb. Vasc. Biol..

[90] Kocken J.M.M., da Costa Martins P.A. (2020). Epigenetic Regulation of Pulmonary Arterial Hypertension-Induced Vascular and Right Ventricular Remodeling: New Opportunities?. Int. J. Mol. Sci..

[91] Saygin D., Tabib T., Bittar H.E.T., Valenzi E., Sembrat J., Chan S.Y., Rojas M., Lafyatis R. (2020). Transcriptional profiling of lung cell populations in idiopathic pulmonary arterial hypertension. Pulm. Circ..

[92] Yu Q., Tai Y.Y., Tang Y., Zhao J., Negi V., Culley M.K., Pilli J., Sun W., Brugger K., Mayr J. (2019). BOLA (BolA Family Member 3) Deficiency Controls Endothelial Metabolism and Glycine Homeostasis in Pulmonary Hypertension. Circulation.

[93] Reyes-Palomares A., Gu M., Grubert F., Berest I., Sa S., Kasowski M., Arnold C., Shuai M., Srivas R., Miao S. (2020). Remodeling of active endothelial enhancers is associated with aberrant gene-regulatory networks in pulmonary arterial hypertension. Nat. Commun..

[94] Li Y., Liu S., Zhang Y., Gao Q., Sun W., Fu L., Cao J. (2018). Histone demethylase JARID1B regulates proliferation and migration of pulmonary arterial smooth muscle cells in mice with chronic hypoxia-induced pulmonary hypertension via nuclear factor-kappa B (NFkB). Cardiovasc. Pathol..

[95] Ozinsky A., Underhill D.M., Fontenot J.D., Hajjar A.M., Smith K.D., Wilson C.B., Schroeder L., Aderem A. (2000). The repertoire for pattern recognition of pathogens by the innate immune system is defined by cooperation between toll-like receptors. Proc. Natl. Acad. Sci. USA.

[96] Akira S., Uematsu S., Takeuchi O. (2006). Pathogen recognition and innate immunity. Cell.

[97] Schiopu A., Cotoi O.S. (2013). S100A8 and S100A9: DAMPs at the crossroads between innate immunity, traditional risk factors, and cardiovascular disease. Mediators Inflamm..

[98] Tang D., Kang R., Coyne C.B., Zeh H.J., Lotze M.T. (2012). PAMPs and DAMPs: Signal 0s that spur autophagy and immunity. Immunol. Rev..

[99] Chen T., Zhang X., Zhu G., Liu H., Chen J., Wang Y., He X. (2020). Quercetin inhibits TNF-α induced HUVECs apoptosis and inflammation via downregulating NF-kB and AP-1 signaling pathway in vitro. Medicine.

[100] Jiang M., Wang H., Liu Z., Lin L., Wang L., Xie M., Li D., Zhang J., Zhang R. (2020). Endoplasmic reticulum stress-dependent activation of iNOS/NO-NF-κB signaling and NLRP3 inflammasome contributes to endothelial inflammation and apoptosis associated with microgravity. FASEB J..

[101] Joffre J., Hellman J., Ince C., Ait-Oufella H. (2020). Endothelial Responses in Sepsis. Am. J. Respir. Crit. Care Med..

[102] Mi L., Zhang Y., Xu Y., Zheng X., Zhang X., Wang Z., Xue M., Jin X. (2019). HMGB1/RAGE pro-inflammatory axis promotes vascular endothelial cell apoptosis in limb ischemia/reperfusion injury. Biomed. Pharmacother..

[103] Shao Y., Saredy J., Xu K., Sun Y., Saaoud F., Drummer C.t., Lu Y., Luo J.J., Lopez-Pastrana J., Choi E.T. (2021). Endothelial Immunity Trained by Coronavirus Infections, DAMP Stimulations and Regulated by Anti-Oxidant NRF2 May Contribute to Inflammations, Myelopoiesis, COVID-19 Cytokine Storms and Thromboembolism. Front. Immunol..

[104] Tseng C.Y., Wang J.S., Chao M.W. (2017). Causation by Diesel Exhaust Particles of Endothelial Dysfunctions in Cytotoxicity, Pro-inflammation, Permeability, and Apoptosis Induced by ROS Generation. Cardiovasc. Toxicol..

[105] Mitchell J.A., Ryffel B., Quesniaux V.F., Cartwright N., Paul-Clark M. (2007). Role of pattern-recognition receptors in cardiovascular health and disease. Biochem. Soc. Trans..

[106] Andersson U., Ottestad W., Tracey K.J. (2020). Extracellular HMGB1: A therapeutic target in severe pulmonary inflammation including COVID-19?. Mol. Med..

[107] Amarante-Mendes G.P., Adjemian S., Branco L.M., Zanetti L.C., Weinlich R., Bortoluci K.R. (2018). Pattern Recognition Receptors and the Host Cell Death Molecular Machinery. Front. Immunol..

[108] Escamilla-Tilch M., Filio-Rodríguez G., García-Rocha R., Mancilla-Herrera I., Mitchison N.A., Ruiz-Pacheco J.A., Sánchez-García F.J., Sandoval-Borrego D., Vázquez-Sánchez E.A. (2013). The interplay between pathogen-associated and danger-associated molecular patterns: An inflammatory code in cancer?. Immunol. Cell. Biol..

[109] McCall K.D., Muccioli M., Benencia F. (2020). Toll-Like Receptors Signaling in the Tumor Microenvironment. Adv. Exp. Med. Biol..

[110] Medzhitov R., Preston-Hurlburt P., Janeway C.A. (1997). A human homologue of the Drosophila Toll protein signals activation of adaptive immunity. Nature.

[111] Miettinen M., Sareneva T., Julkunen I., Matikainen S. (2001). IFNs activate toll-like receptor gene expression in viral infections. Genes. Immun..

[112] Alexopoulou L., Holt A.C., Medzhitov R., Flavell R.A. (2001). Recognition of double-stranded RNA and activation of NF-kappaB by Toll-like receptor 3. Nature.

[113] George P.M., Badiger R., Shao D., Edwards M.R., Wort S.J., Paul-Clark M.J., Mitchell J.A. (2012). Viral Toll Like Receptor activation of pulmonary vascular smooth muscle cells results in endothelin-1 generation; relevance to pathogenesis of pulmonary arterial hypertension. Biochem. Biophys. Res. Commun..

[114] Yanagisawa M., Kurihara H., Kimura S., Tomobe Y., Kobayashi M., Mitsui Y., Yazaki Y., Goto K., Masaki T. (1988). A novel potent vasoconstrictor peptide produced by vascular endothelial cells. Nature.

[115] Kawai T., Akira S. (2010). The role of pattern-recognition receptors in innate immunity: Update on Toll-like receptors. Nat. Immunol..

[116] Karikó K., Ni H., Capodici J., Lamphier M., Weissman D. (2004). mRNA is an endogenous ligand for Toll-like receptor 3. J. Biol. Chem..

[117] Bianchi F., Alexiadis S., Camisaschi C., Truini M., Centonze G., Milione M., Balsari A., Tagliabue E., Sfondrini L. (2020). TLR3 Expression Induces Apoptosis in Human Non-Small-Cell Lung Cancer. Int. J. Mol. Sci..

[118] Gao T., Zhang S.P., Wang J.F., Liu L., Wang Y., Cao Z.Y., Hu Q.K., Yuan W.J., Lin L. (2018). TLR3 contributes to persistent autophagy and heart failure in mice after myocardial infarction. J. Cell. Mol. Med..

[119] Cole J.E., Navin T.J., Cross A.J., Goddard M.E., Alexopoulou L., Mitra A.T., Davies A.H., Flavell R.A., Feldmann M., Monaco C. (2011). Unexpected protective role for Toll-like receptor 3 in the arterial wall. Proc. Natl. Acad. Sci. USA.

[120] Farkas D., Thompson A.A.R., Bhagwani A.R., Hultman S., Ji H., Kotha N., Farr G., Arnold N.D., Braithwaite A., Casbolt H. (2019). Toll-like Receptor 3 Is a Therapeutic Target for Pulmonary Hypertension. Am. J. Respir. Crit. Care Med..

[121] Zimmer S., Steinmetz M., Asdonk T., Motz I., Coch C., Hartmann E., Barchet W., Wassmann S., Hartmann G., Nickenig G. (2011). Activation of endothelial toll-like receptor 3 impairs endothelial function. Circ. Res..

[122] Bauer E.M., Chanthaphavong R.S., Sodhi C.P., Hackam D.J., Billiar T.R., Bauer P.M. (2014). Genetic deletion of toll-like receptor 4 on platelets attenuates experimental pulmonary hypertension. Circ. Res..

[123] Bauer E.M., Shapiro R., Zheng H., Ahmad F., Ishizawar D., Comhair S.A., Erzurum S.C., Billiar T.R., Bauer P.M. (2013). High mobility group box 1 contributes to the pathogenesis of experimental pulmonary hypertension via activation of Toll-like receptor 4. Mol. Med..

[124] Dai M., Xiao R., Cai L., Ge T., Zhu L., Hu Q. (2019). HMGB1 is mechanistically essential in the development of experimental pulmonary hypertension. Am. J. Physiol. Cell. Physiol..

[125] Ma L., Ambalavanan N., Liu H., Sun Y., Jhala N., Bradley W.E., Dell’Italia L.J., Michalek S., Wu H., Steele C. (2016). TLR4 regulates pulmonary vascular homeostasis and remodeling via redox signaling. Front. Biosci..

[126] Wang J., Tian X.T., Peng Z., Li W.Q., Cao Y.Y., Li Y., Li X.H. (2019). HMGB1/TLR4 promotes hypoxic pulmonary hypertension via suppressing BMPR2 signaling. Vascul. Pharmacol..

[127] Young K.C., Hussein S.M., Dadiz R., deMello D., Devia C., Hehre D., Suguihara C. (2010). Toll-like receptor 4-deficient mice are resistant to chronic hypoxia-induced pulmonary hypertension. Exp. Lung Res..

[128] Zemskova M., McClain N., Niihori M., Varghese M.V., James J., Rafikov R., Rafikova O. (2020). Necrosis-Released HMGB1 (High Mobility Group Box 1) in the Progressive Pulmonary Arterial Hypertension Associated With Male Sex. Hypertension.

[129] Zuo Z.T., Ma Y., Sun Y., Bai C.Q., Zhou H.Y., Chen B.H. (2020). Role of TLR4/NF-κB Signalling Pathway in Pulmonary Arterial Hypertension in Patients with Chronic Obstructive Pulmonary Disease. J. Coll. Physicians Surg. Pak..

[130] Bevilacqua M.P., Pober J.S., Mendrick D.L., Cotran R.S., Gimbrone M.A. (1987). Identification of an inducible endothelial-leukocyte adhesion molecule. Proc. Natl. Acad. Sci. USA.

[131] Dias S., Choy M., Rafii S. (2001). The role of CXC chemokines in the regulation of tumor angiogenesis. Cancer Investig..

[132] Keane M.P., Strieter R.M. (1999). The role of CXC chemokines in the regulation of angiogenesis. Chem. Immunol..

[133] Ridiandries A., Tan J.T., Bursill C.A. (2016). The Role of CC-Chemokines in the Regulation of Angiogenesis. Int. J. Mol. Sci..

[134] Zegeye M.M., Lindkvist M., Fälker K., Kumawat A.K., Paramel G., Grenegård M., Sirsjö A., Ljungberg L.U. (2018). Activation of the JAK/STAT3 and PI3K/AKT pathways are crucial for IL-6 trans-signaling-mediated pro-inflammatory response in human vascular endothelial cells. Cell Commun. Signal..

[135] Lee K., Na W., Lee J.Y., Na J., Cho H., Wu H., Yune T.Y., Kim W.S., Ju B.G. (2012). Molecular mechanism of Jmjd3-mediated interleukin-6 gene regulation in endothelial cells underlying spinal cord injury. J. Neurochem..

[136] Lawrence T. (2009). The nuclear factor NF-kappaB pathway in inflammation. Cold Spring Harb. Perspect. Biol..

[137] Liu J., Li J., Xie C., Xuan L., Tang B. (2020). MSCs attenuate hypoxia induced pulmonary hypertension by activating P53 and NF-kB signaling pathway through TNFα secretion. Biochem. Biophys. Res. Commun..

[138] Sarada S.K.S., Himadri P., Mathew T., Saumya S., Chitharanjan M. (2012). Nifedipine inhibits hypoxia induced transvascular leakage through down regulation of NFkB. Respir. Physiol. Neurobiol..

[139] Bai Y., Li Z.X., Wang H.L., Lian G.C., Wang Y. (2017). The protective effects of PCPA against monocrotaline-induced pulmonary arterial hypertension are mediated through the downregulation of NFAT-1 and NF-κB. Int. J. Mol. Med..

[140] Fan J., Fan X., Li Y., Ding L., Zheng Q., Guo J., Xia D., Xue F., Wang Y., Liu S. (2016). Chronic Normobaric Hypoxia Induces Pulmonary Hypertension in Rats: Role of NF-κB. High Alt. Med. Biol..

[141] Farkas D., Alhussaini A.A., Kraskauskas D., Kraskauskiene V., Cool C.D., Nicolls M.R., Natarajan R., Farkas L. (2014). Nuclear factor κB inhibition reduces lung vascular lumen obliteration in severe pulmonary hypertension in rats. Am. J. Respir. Cell Mol. Biol..

[142] Huang J., Kaminski P.M., Edwards J.G., Yeh A., Wolin M.S., Frishman W.H., Gewitz M.H., Mathew R. (2008). Pyrrolidine dithiocarbamate restores endothelial cell membrane integrity and attenuates monocrotaline-induced pulmonary artery hypertension. Am. J. Physiol. Lung Cell. Mol. Physiol..

[143] Price L.C., Caramori G., Perros F., Meng C., Gambaryan N., Dorfmuller P., Montani D., Casolari P., Zhu J., Dimopoulos K. (2013). Nuclear factor κ-B is activated in the pulmonary vessels of patients with end-stage idiopathic pulmonary arterial hypertension. PLoS ONE.

[144] Cheng M.H., Wong Y.H., Chang C.M., Yang C.C., Chen S.H., Yuan C.L., Kuo H.M., Yang C.Y., Chiu H.F. (2017). B1, a novel HDAC inhibitor, induces apoptosis through the regulation of STAT3 and NF-κB. Int. J. Mol. Med..

[145] Huang X., Gong R., Li X., Virtue A., Yang F., Yang I.H., Tran A.H., Yang X.F., Wang H. (2013). Identification of novel pretranslational regulatory mechanisms for NF-κB activation. J. Biol. Chem..

[146] Liu D., Perkins J.T., Hennig B. (2016). EGCG prevents PCB-126-induced endothelial cell inflammation via epigenetic modifications of NF-κB target genes in human endothelial cells. J. Nutr. Biochem..

[147] Liu D., Perkins J.T., Petriello M.C., Hennig B. (2015). Exposure to coplanar PCBs induces endothelial cell inflammation through epigenetic regulation of NF-κB subunit p65. Toxicol. Appl. Pharmacol..

[148] Song L., Lin C., Gong H., Wang C., Liu L., Wu J., Tao S., Hu B., Cheng S.Y., Li M. (2013). miR-486 sustains NF-κB activity by disrupting multiple NF-κB-negative feedback loops. Cell Res..

[149] Yu S., Chen X., Xiu M., He F., Xing J., Min D., Guo F. (2017). The regulation of Jmjd3 upon the expression of NF-κB downstream inflammatory genes in LPS activated vascular endothelial cells. Biochem. Biophys. Res. Commun..

[150] Andrilenas K.K., Ramlall V., Kurland J., Leung B., Harbaugh A.G., Siggers T. (2018). DNA-binding landscape of IRF3, IRF5 and IRF7 dimers: Implications for dimer-specific gene regulation. Nucleic. Acids. Res..

[151] Canivet C., Rhéaume C., Lebel M., Piret J., Gosselin J., Boivin G. (2018). Both IRF3 and especially IRF7 play a key role to orchestrate an effective cerebral inflammatory response in a mouse model of herpes simplex virus encephalitis. J. Neurovirol..

[152] Cheng Y., Zhu W., Ding C., Niu Q., Wang H., Yan Y., Sun J. (2019). IRF7 Is Involved in Both STING and MAVS Mediating IFN-β Signaling in IRF3-Lacking Chickens. J. Immunol..

[153] Simons K.H., de Vries M.R., de Jong R.C.M., Peters H.A.B., Jukema J.W., Quax P.H.A. (2019). IRF3 and IRF7 mediate neovascularization via inflammatory cytokines. J. Cell. Mol. Med..

[154] Bhagwani A., Thompson A.A.R., Farkas L. (2020). When Innate Immunity Meets Angiogenesis-The Role of Toll-Like Receptors in Endothelial Cells and Pulmonary Hypertension. Front. Med..

[155] Turton H.A., Thompson A.A.R., Farkas L. (2020). RNA Signaling in Pulmonary Arterial Hypertension-A Double-Stranded Sword. Int. J. Mol. Sci..

[156] Demerouti E., Karyofyllis P., Athanassopoulos G., Karatasakis G., Tsiapras D., Manginas A., Voudris V. (2019). Pulmonary arterial hypertension associated with interferon-beta treatment for multiple sclerosis. Case report and literature review. Mult. Scler. Relat. Disord..

[157] Fok A., Williams T., McLean C.A., Butler E. (2016). Interferon beta-1a long-term therapy related to pulmonary arterial hypertension in multiple sclerosis patients. Mult. Scler..

[158] Lerche M., Eichstaedt C.A., Hinderhofer K., Grünig E., Tausche K., Ziemssen T., Halank M., Wirtz H., Seyfarth H.J. (2019). Mutually reinforcing effects of genetic variants and interferon-β 1a therapy for pulmonary arterial hypertension development in multiple sclerosis patients. Pulm. Circ..

[159] Bauer E.M., Zheng H., Lotze M.T., Bauer P.M. (2014). Recombinant human interferon alpha 2b prevents and reverses experimental pulmonary hypertension. PLoS ONE.

[160] Das A., Chai J.C., Kim S.H., Lee Y.S., Park K.S., Jung K.H., Chai Y.G. (2015). Transcriptome sequencing of microglial cells stimulated with TLR3 and TLR4 ligands. BMC Genom..

[161] Lai Q., Wang H., Li A., Xu Y., Tang L., Chen Q., Zhang C., Gao Y., Song J., Du Z. (2018). Decitibine improve the efficiency of anti-PD-1 therapy via activating the response to IFN/PD-L1 signal of lung cancer cells. Oncogene.

[162] De Pascali F., Hemann C., Samons K., Chen C.A., Zweier J.L. (2014). Hypoxia and reoxygenation induce endothelial nitric oxide synthase uncoupling in endothelial cells through tetrahydrobiopterin depletion and S-glutathionylation. Biochemistry.

[163] Makarenko V.V., Usatyuk P.V., Yuan G., Lee M.M., Nanduri J., Natarajan V., Kumar G.K., Prabhakar N.R. (2014). Intermittent hypoxia-induced endothelial barrier dysfunction requires ROS-dependent MAP kinase activation. Am. J. Physiol.-Cell Physiol..

[164] Mojiri A., Nakhaii-Nejad M., Phan W.L., Kulak S., Radziwon-Balicka A., Jurasz P., Michelakis E., Jahroudi N. (2013). Hypoxia results in upregulation and de novo activation of von Willebrand factor expression in lung endothelial cells. Arterioscler. Thromb. Vasc. Biol..

[165] Tremblay J.C., Ainslie P.N., Turner R., Gatterer H., Schlittler M., Woyke S., Regli I.B., Strapazzon G., Rauch S., Siebenmann C. (2020). Endothelial function and shear stress in hypobaric hypoxia: Time course and impact of plasma volume expansion in men. Am. J. Physiol. Heart Circ. Physiol..

[166] Tymko M.M., Tremblay J.C., Bailey D.M., Green D.J., Ainslie P.N. (2019). The impact of hypoxaemia on vascular function in lowlanders and high altitude indigenous populations. J. Physiol..

[167] Zhang J., Hu H., Palma N.L., Harrison J.K., Mubarak K.K., Carrie R.D., Alnuaimat H., Shen X., Luo D., Patel J.M. (2012). Hypoxia-induced endothelial CX3CL1 triggers lung smooth muscle cell phenotypic switching and proliferative expansion. Am. J. Physiol. Lung Cell. Mol. Physiol..

[168] Jiang B.H., Semenza G.L., Bauer C., Marti H.H. (1996). Hypoxia-inducible factor 1 levels vary exponentially over a physiologically relevant range of O2 tension. Am. J. Physiol..

[169] Wilson J.W., Shakir D., Batie M., Frost M., Rocha S. (2020). Oxygen-sensing mechanisms in cells. FEBS J..

[170] Bartoszewski R., Moszyńska A., Serocki M., Cabaj A., Polten A., Ochocka R., Dell’Italia L., Bartoszewska S., Króliczewski J., Dąbrowski M. (2019). Primary endothelial cell-specific regulation of hypoxia-inducible factor (HIF)-1 and HIF-2 and their target gene expression profiles during hypoxia. FASEB J..

[171] Tang H., Babicheva A., McDermott K.M., Gu Y., Ayon R.J., Song S., Wang Z., Gupta A., Zhou T., Sun X. (2018). Endothelial HIF-2α contributes to severe pulmonary hypertension due to endothelial-to-mesenchymal transition. Am. J. Physiol. Lung Cell. Mol. Physiol..

[172] Carmeliet P., Dor Y., Herbert J.M., Fukumura D., Brusselmans K., Dewerchin M., Neeman M., Bono F., Abramovitch R., Maxwell P. (1998). Role of HIF-1alpha in hypoxia-mediated apoptosis, cell proliferation and tumour angiogenesis. Nature.

[173] Papandreou I., Cairns R.A., Fontana L., Lim A.L., Denko N.C. (2006). HIF-1 mediates adaptation to hypoxia by actively downregulating mitochondrial oxygen consumption. Cell Metab..

[174] Pugh C.W., Ratcliffe P.J. (2003). Regulation of angiogenesis by hypoxia: Role of the HIF system. Nat. Med..

[175] Forsythe J.A., Jiang B.H., Iyer N.V., Agani F., Leung S.W., Koos R.D., Semenza G.L. (1996). Activation of vascular endothelial growth factor gene transcription by hypoxia-inducible factor 1. Mol. Cell. Biol..

[176] Kimura H., Esumi H. (2003). Reciprocal regulation between nitric oxide and vascular endothelial growth factor in angiogenesis. Acta Biochim. Pol..

[177] Fijalkowska I., Xu W., Comhair S.A., Janocha A.J., Mavrakis L.A., Krishnamachary B., Zhen L., Mao T., Richter A., Erzurum S.C. (2010). Hypoxia inducible-factor1alpha regulates the metabolic shift of pulmonary hypertensive endothelial cells. Am. J. Pathol..

[178] Frump A.L., Selej M., Wood J.A., Albrecht M., Yakubov B., Petrache I., Lahm T. (2018). Hypoxia Upregulates Estrogen Receptor β in Pulmonary Artery Endothelial Cells in a HIF-1α-Dependent Manner. Am. J. Respir. Cell Mol. Biol..

[179] He M., Ma S., Cai Q., Wu Y., Shao C., Kong H., Wang H., Zeng X., Xie W. (2018). Hypoxia induces the dysfunction of human endothelial colony-forming cells via HIF-1α signaling. Respir. Physiol. Neurobiol..

[180] Makker K., Afolayan A.J., Teng R.J., Konduri G.G. (2019). Altered hypoxia-inducible factor-1α (HIF-1α) signaling contributes to impaired angiogenesis in fetal lambs with persistent pulmonary hypertension of the newborn (PPHN). Physiol. Rep..

[181] Dai Z., Zhu M.M., Peng Y., Machireddy N., Evans C.E., Machado R., Zhang X., Zhao Y.Y. (2018). Therapeutic Targeting of Vascular Remodeling and Right Heart Failure in Pulmonary Arterial Hypertension with a HIF-2α Inhibitor. Am. J. Respir. Crit. Care Med..

[182] Labrousse-Arias D., Castillo-González R., Rogers N.M., Torres-Capelli M., Barreira B., Aragonés J., Cogolludo Á., Isenberg J.S., Calzada M.J. (2016). HIF-2α-mediated induction of pulmonary thrombospondin-1 contributes to hypoxia-driven vascular remodelling and vasoconstriction. Cardiovasc. Res..

[183] Sun X., Sun B.L., Babicheva A., Vanderpool R., Oita R.C., Casanova N., Tang H., Gupta A., Lynn H., Gupta G. (2020). Direct Extracellular NAMPT Involvement in Pulmonary Hypertension and Vascular Remodeling. Transcriptional Regulation by SOX and HIF-2α. Am. J. Respir. Cell Mol. Biol..

[184] Ranchoux B., Antigny F., Rucker-Martin C., Hautefort A., Péchoux C., Bogaard H.J., Dorfmüller P., Remy S., Lecerf F., Planté S. (2015). Endothelial-to-mesenchymal transition in pulmonary hypertension. Circulation.

[185] Suzuki T., Carrier E.J., Talati M.H., Rathinasabapathy A., Chen X., Nishimura R., Tada Y., Tatsumi K., West J. (2018). Isolation and characterization of endothelial-to-mesenchymal transition cells in pulmonary arterial hypertension. Am. J. Physiol. Lung Cell. Mol. Physiol..

[186] Zhao L., Chen C.N., Hajji N., Oliver E., Cotroneo E., Wharton J., Wang D., Li M., McKinsey T.A., Stenmark K.R. (2012). Histone deacetylation inhibition in pulmonary hypertension: Therapeutic potential of valproic acid and suberoylanilide hydroxamic acid. Circulation.

[187] Zhou X.L., Huang F.J., Li Y., Huang H., Wu Q.C. (2021). SEDT2/METTL14-mediated m6A methylation awakening contributes to hypoxia-induced pulmonary arterial hypertension in mice. Aging.

[188] Archer S.L., Marsboom G., Kim G.H., Zhang H.J., Toth P.T., Svensson E.C., Dyck J.R., Gomberg-Maitland M., Thébaud B., Husain A.N. (2010). Epigenetic attenuation of mitochondrial superoxide dismutase 2 in pulmonary arterial hypertension: A basis for excessive cell proliferation and a new therapeutic target. Circulation.

[189] Li M., Riddle S.R., Frid M.G., El Kasmi K.C., McKinsey T.A., Sokol R.J., Strassheim D., Meyrick B., Yeager M.E., Flockton A.R. (2011). Emergence of fibroblasts with a proinflammatory epigenetically altered phenotype in severe hypoxic pulmonary hypertension. J. Immunol..

